# The impact of socioeconomic and stimulus inequality on human brain physiology

**DOI:** 10.1038/s41598-021-85236-z

**Published:** 2021-04-02

**Authors:** Dhanya Parameshwaran, S. Sathishkumar, Tara C. Thiagarajan

**Affiliations:** Sapien Labs, 1201 Wilson Drive 27th Floor, Arlington, VA 22209 USA

**Keywords:** Neuroscience, Physiology, Systems biology

## Abstract

The brain undergoes profound structural and dynamical alteration in response to its stimulus environment. In animal studies, enriched stimulus environments result in numerous structural and dynamical changes along with cognitive enhancements. In human society factors such as education, travel, cell phones and motorized transport dramatically expand the rate and complexity of stimulus experience but diverge in access based on income. Correspondingly, poverty is associated with significant structural and dynamical differences in the brain, but it is unknown how this relates to disparity in stimulus access. Here we studied consumption of major stimulus factors along with measurement of brain signals using EEG in 402 people in India across an income range of $0.82 to $410/day. We show that the complexity of the EEG signal scaled logarithmically with overall stimulus consumption and income and linearly with education and travel. In contrast phone use jumped up at a threshold of $30/day corresponding to a similar jump in key spectral parameters that reflect the signal energy. Our results suggest that key aspects of brain physiology increase in lockstep with stimulus consumption and that we have not fully appreciated the profound way that stimulus expanding aspects of modern life are changing our brain physiology.

## Introduction

Universal education based on literacy and numeracy, and technologies such as electricity, telecommunications and motorized transport are relatively recent advances in human society that expand the rate and scope of stimulus experience. Education expands the scope of knowledge encountered, and transport increases the rate of visual stimulus, the extent of spatial environment encountered in a day and travel to novel spatial environments, languages and cultures, while phone use expands social interaction and access to information of various kinds. However large populations still lack formal education and literacy, walk long distances to access public transport, and still do not have smart phones. What is the impact of this profound divergence in socioeconomic and stimulus environment on human brain physiology?

This is a fundamentally important question because unlike any other organ, the brain is experience dependent in its development and function; in addition to consuming nutrients, it must ‘consume’ stimulus to inform its growth^1–3^. Placing rodents in a more complex environment has far reaching impact on gene expression in the brain^4^, the degree and nature of synaptic plasticity^5–8^, branching of neuronal dendrites^9,10^, brain surface area and a host of other functional and structural aspects^11,12^ and reduces the negative impact of various disorders^13^. Correspondingly, studies demonstrate that childhood poverty in the United States, which impacts access to stimulus, has a dramatic negative effect on dynamical and structural elements of the brain such as cortical volume^14^ and surface area^15^, as well as gamma power in the EEG^16^. Conversely years of education correlates positively with these structural aspects of the cortex^17,18^.

In this study we probe the relationship between key aspects of human brain physiology and major stimulus-expanding factors, contrasting it to the relationship to consumption of major food groups (diet factors). To measure brain activity, we used electroencephalography or EEG which records electrical activity using noninvasive electrodes placed on the scalp. We then quantified the complexity and energy of the signal, both key measures of brain activity with significant implications for cognitive function and health. Complexity represents the diversity of patterns produced in the signal and has been associated with performance on a pattern recognition task^19^ and may correlate with states of consciousness and alertness^20–22^. Energy was assessed with multiple metrics derived from the power spectrum. The alpha oscillation, a key spectral or energy measure, positively correlates with various cognitive processes such as working memory and attention^23^ where it is hypothesized to play a role in suppression of distracters to enable selective attention and mental imagery^24–26^.

This study, carried out in Tamil Nadu India, compared key features of resting EEG activity in adults with a wide range of household income and stimulus consumption patterns with respect to education, travel, mobile phone usage, electricity and fuel consumption.

Broadly, our results show a systematic increase in both energy and complexity of the signal with income, that occurred in lockstep with increasing stimulus consumption, resulting in a several fold difference between the lowest and highest stimulus consumption groups. Our data suggest that whereas phone usage may be the major driver of differences in signal energy, travel and education enhance complexity of the signal. These findings have enormous implications for how we must understand and approach brain health in a global context.

## Materials and methods

### Sampling

402 participants between the ages of 21 and 65 were recruited in Tamil Nadu, India from locations that spanned a range of literacy levels and infrastructural features such as distance to road and electrification. This included remote settlements with little to no access to electricity, phones or motorized transport to a city of several million people with all modern resources and amenities. 16% of participants came from villages with populations less than 2000 with no secondary education facilities, public transport or telecommunication, 50% came from villages and towns ranging from 5000 to 10,000 people with a diversity of infrastructure, 12% were from towns with populations of ~ 100,000 and 22% came from million person cities with international airports. In each location participants were selected to span as evenly as possible each age decade from 20 to 60 s, and within each age band were split roughly equally by gender. Participants were also recruited across a breadth of household income in each ecosystem. Annual incomes from $300 to approximately $150,000 dollars (~ $0.85 to $410/day). In the smaller villages this spanned $0.85 to ~ $5 per day while in the cities it ranged from $2.50 to ~ $410/day and within each income band ($0–$10/day, $10–$30/day and > $30/day) were spread roughly equally by age and gender. Education levels spanned from no schooling to college or beyond.

### Participant recruitment

Interested participants in each location were fully informed about the intent of the experiment, provided with demonstrations of the recording and experimental process and asked to sign a consent form. All recruitment, consent and data collection were carried out in accordance with protocols approved by Health Media IRB (USA, OHRP IRB #00001211) and Sigma-IRB (India) in accordance with Title 45, code of federal regulations, sub-part A of NIH (USA) and Indian Independent Ethics Committee requirements. Participants were then surveyed to determine if they were appropriate for inclusion based on our sampling criteria of age, gender and income and had no known history of physical or mental illness. If so, a time was scheduled for the data collection which included surveys of their demographics, diet, technology use and travel behavior along with EEG recordings. Participants were instructed to wash and dry their hair on the day of the EEG recording without the application of hair products, particularly hair oil. Low-income participants were provided with a sachet of shampoo. Participants were excluded from participation if they reported any illness (headache, nausea, cough, cold) on the day of the recording. Recordings were carried out indoors, typically in the home of the participant or sometimes in public locations such as offices, village halls or schools. Care was taken to select locations at distance from noise producing equipment such as mobile towers, electric motors and pumps. Low-income participants were paid Rs. 150 ($2.50) to compensate for potential loss of wages.

### Survey of demographics, stimulus and diet

Survey data was collected on a tablet using forms with check boxes, radio buttons and drop downs to minimize data ambiguities. Details of data captured is provided in Table 1 (full questionnaire in Supplementary Materials; Distributions in Supplementary Fig. 1; Supplementary Table 1). In the case of travel and diet, answers were coded into ordinal groups for simplicity (into four travel distance categories and three groups of frequency of consumption for diet). In aggregate, out of the total sample, the diet questions were administered to 370. In a few cases participants did not know an answer to a question or occasionally declined to answer.

**Table 1:** Description of survey elements.

	Survey elements	Description
1	Population	Population of settlement where individual is residing as per government census of associated revenue village. If location is greater than 2 km from revenue village, hamlet population obtained from panchayat (local government) is used
2	Household income	Acquired as total monthly household income in Indian Rupees and converted to USD at the exchange rate at the time of data acquisition of Rs 60 per USD and then represented in income *per day*. A monthly income of 54,000 Rupees per month translates to $30 per day
**Stimulus factors**		
3	Education level	Number of years of completed education counting from grade 1. Of those who attended college, all in the sample completed it. Note all college graduates are marked as 16 years of education, although some attended 3-year colleges. No. of years of education beyond 16 was not noted. However 12 members of the sample had > 16 years of education
4	Farthest travel (past year)	Farthest distance traveled from home in the one year before the date of survey. Specific locations were noted by asking respondents to list the furthest places they had been and then coded by the following categories: The highest coded location was used 1: Within home town 2: Within 100 km from hometown in the same State (typically a day trip) 3: > 100 km from hometown in the Same State (typically requires overnight stay) 4: Within 100 km from hometown to a different State (note that State boundaries in India are based on language so different State indicates a different language) 5: > 100 km from hometown to a different State 6: To a different country
5	Farthest travel (lifetime)	Farthest location from their current home that they have ever been in their life for any reason at any age. Locations were coded for analysis by the same categories as above
7	Fuel consumption	This refers to the amount they spent on purchasing petrol or gasoline in the previous full month shown in Indian Rupees. The price of petrol was ~ Rs. 75/liter or $4.75/ gallon at the time of survey. The range of fuel purchase ranged from 0 (no vehicle) or going from 1 L all the way to 160 L or 42 gallons, translating to miles traveled in the month of anywhere from 20 to 1500 for one person in the transportation business
8	Electricity usage	This refers to the amount they spent on household electricity in the previous full month shown in Indian Rupees. The price of electricity at the time of survey ranged from Rs. 1 to Rs. 4 per unit or kWH. The tariff was a sliding scale with lower prices for those consuming lower levels of electricity. Consequently an exact conversion to units consumed was not readily possible
9	Phone usage	This refers to the amount spent on phone communication or other usage in the previous full month shown in Indian Rupees converted to USD at 60 Rs. per USD. Note that due to various pricing schemes of prepaid SIM cards as well as data plans it was not possible to convert these numbers to any particular talk time or data usage
**Diet factors**		
11	Grains frequency	Average frequency of consumption of major grain-based foods in the region (rice, wheat and millet based). Respondents were asked how frequently they consumed each food per week and answers were categorized as follows: once or less in the past month, 1–3 times per week, more than 4 times per week. Categories were given weightings of 0.1, 2 and 5 respectively. Frequency refers to the average of the selected category weightings across all foods in the food group
12	Grains variety	Total consumption of all major grain-based foods in the region computed by summing the category weightings selected across all foods in the food group
13	Protein frequency	Average frequency of consumption of major protein-based foods in the region (meat, chicken, eggs, milk, yoghurt) computed as in 11
14	Protein variety	Total consumption of major protein-based foods in the region (meat, chicken, eggs, milk, yoghurt) computed as in 11
15	Vegetable frequency	Average frequency of consumption of 21 major types of vegetables in the region computed as in 11
16	Vegetable variety	Total consumption of 21 major types of vegetables in the region computed as in 11
17	Fruit frequency	Average frequency of consumption of 15 major types of fruits available in the region computed as in 11
18	Fruit variety	Total consumption of 15 major types of fruits available in the region computed as in 11

### EEG recordings

Resting state EEG activity was captured for 3–4 min when the subject was sitting still with their eyes closed using the 14 channel Emotiv EPOC EEG headset (14 gold plated electrodes and 2 reference electrodes (M1, a ground reference point for measuring the voltage of the other sensors and M2, a feed-forward reference point for reducing electrical interference from external sources). The Emotiv EPOC is an inexpensive, portable and easy to use device, making it very advantageous for large-scale studies across multiple locations. In addition, it has a 12-h battery life which is convenient for recording in remote locations where electricity may be absent or intermittent. The raw signal had an internal sampling rate of 2048 Hz that was digitized at 128 Hz. It was then filtered with a digital 5th order Sinc filter and notch filters at 50 and 60 Hz with a resulting effective bandwidth of 0.2 and 45 Hz. Channels where Emotiv’s internal channel quality metric (based on channel impedance estimates) was less than 5 for 90% of the recording were discarded.

### Computation of EEG metrics

We then computed complexity and various aspects of energy of the EEG signal including total alpha power, alpha energy, peak alpha frequency and theta-beta ratio (Distributions in Supplementary Fig. 2; Supplementary Table 2).

*Waveform Complexity*: This complexity metric is a unitless measure of the diversity of waveform shapes in the signal on long timescales and is described in detail in^19^. Briefly it compares the complex shapes of waveforms on a timescale of 500 ms, which is on the order of the time scales of perception to provide a metric of waveform diversity where 100 indicates a maximal diversity of waveform shapes. This complexity metric is correlated with other entropy and complexity metrics such as Spectral Entropy, Sample entropy and Lempel–Ziv complexity which also attempt to quantify diversity and information content of the signal. However it contrasts to entropy measures such as Permutation Entropy and Sample Entropy, which typically utilize very short time scales or embedding dimensions on the order of a few milliseconds, and/or do not consider the relative amplitude values of the waveforms. It also provides greater discrimination across individuals and has also performed better in predicting results on a pattern recognition task^19^. We note that permutation and sample entropy while directionally similar do not provide significant results in this data (not shown).

### Energy metrics

**Figure 1:**
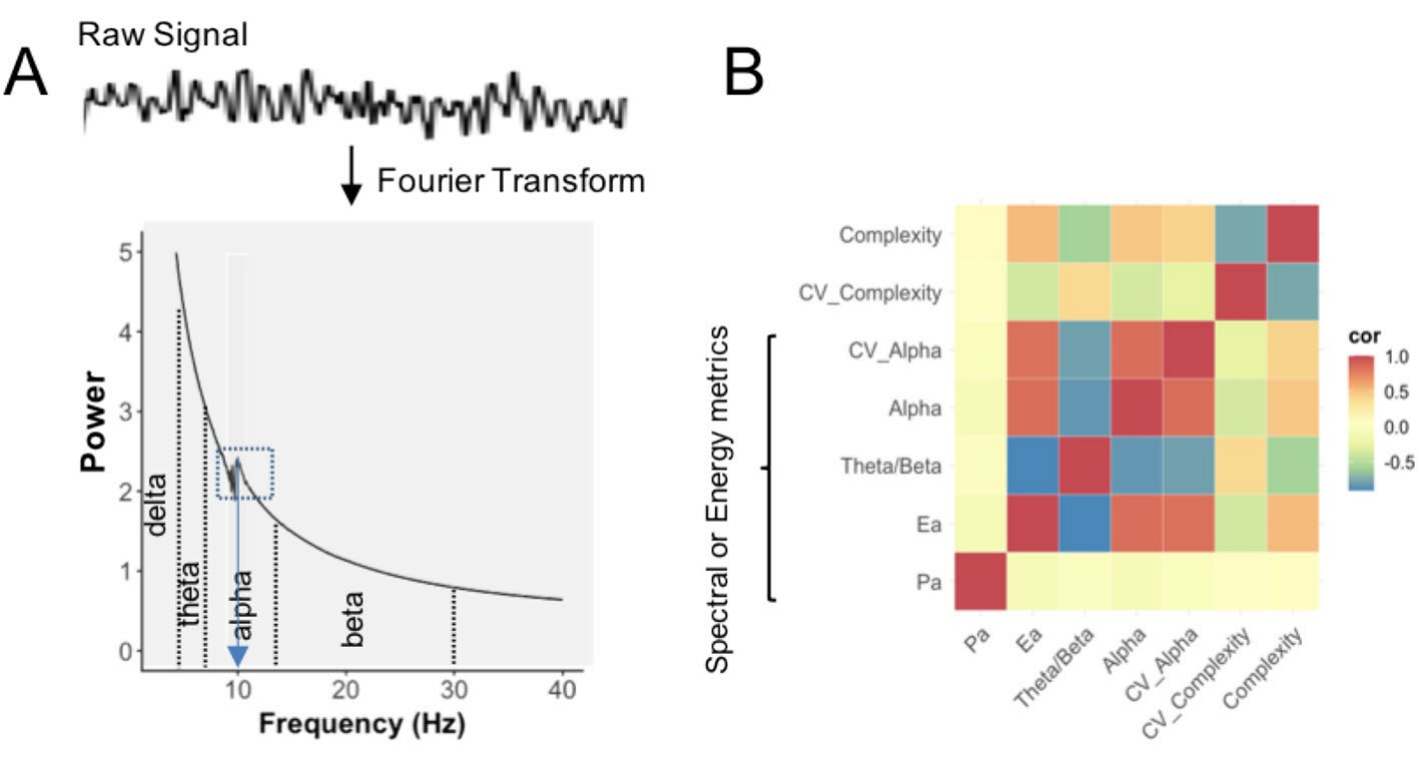
EEG spectral or energy metrics. (**A**) Example of raw signal transformed to a power spectrum showing Alpha band (area under the spectrum labeled alpha), Ealpha (peak highlighted by box), Peak Alpha or Pa (frequency on x-axis corresponding to peak shown by arrow) and Theta-Beta Ratio (ratio of area marked theta divided by area marked beta). (**B**) Correlation of EEG metrics shows spectral or energy metrics other than Pa are highly correlated providing multiple views of a similar aspect of the signal.

Here we use the term energy to refer to greater high frequency components in the signal. This is an approximation for the purpose of easy intuition and not accurate in the strict sense since these metrics are based on the power spectral density (PSD) which refers to the spectral energy distribution per unit time. ‘Energy metrics’ and ‘spectral metrics’ are therefore used interchangeably. The PSD, which involves a transformation of the EEG signal to the spectral domain, was computed for each channel in *R* using the Welch function with a hamming window and 50% overlap. The power was then summed across different frequency bands (Theta (4–7.5 Hz), Alpha (7.5–14 Hz) and Beta (14–30 Hz)) to obtain the total power in each of these bands (Fig. 1A). The alpha band often exhibits a peak above the background decay (marked by box in panel A above) reflecting the presence of an embedded rhythmic oscillation in the signal. Multiple metrics of the PSD were then computed as follows, primarily focusing on the alpha band (histograms of all metrics Supplementary Fig. 2; spatial distribution Supplementary Figs. 3, 4).

Alpha is the relative power in the alpha band calculated by summing the total power across this range of frequency and dividing by the total power across the full range from 0–50 Hz for each channel, and then averaging across channels. It is therefore shown as a fraction and is unitless.

Peak Alpha (Pa) refers to the frequency in Hertz corresponding to the peak seen the alpha band shown by arrow in Fig. 1A and Alpha Energy (Ea, units µV/Hz) is a characterization of the peak, arising above the background decay using the algorithm described in^27^.

CV_Alpha refers to the co-efficient of variance or (SD_alpha_/mean_alpha_)*100, where SD_alpha_ and mean_alpha_ are the standard deviation and mean of the relative alpha power across all channels respectively. This metric is also unitless, as relative alpha power is unitless, and provides a view of the spatial variability of the alpha power across the brain.

Theta-beta ratio was computed as the ratio of the power of the signal within the theta band (4–7.5 Hz) and the beta band (14–30 Hz) calculated for each channel and then averaged across channels. It is unitless and a higher theta-beta ratio, implies a greater relative proportion of lower frequencies or *lower* energy in the signal overall.

All energy (i.e. spectral) metrics except for Pa are significantly correlated with one another (Fig. 1B; *r* > 0.8, *p* < 2.1E−96 for alpha metrics and r <  − 0.79 vs theta-beta, *p* < 1.3E−85; Supplementary Table 3). Complexity is also correlated with spectral metrics but less so (r < 0.47, *p* = 7.6E−18) reflecting the greater potential diversity of waveforms that can be created with higher frequencies, but providing a relatively distinct view.

### Principal component analysis

**Figure 2:**
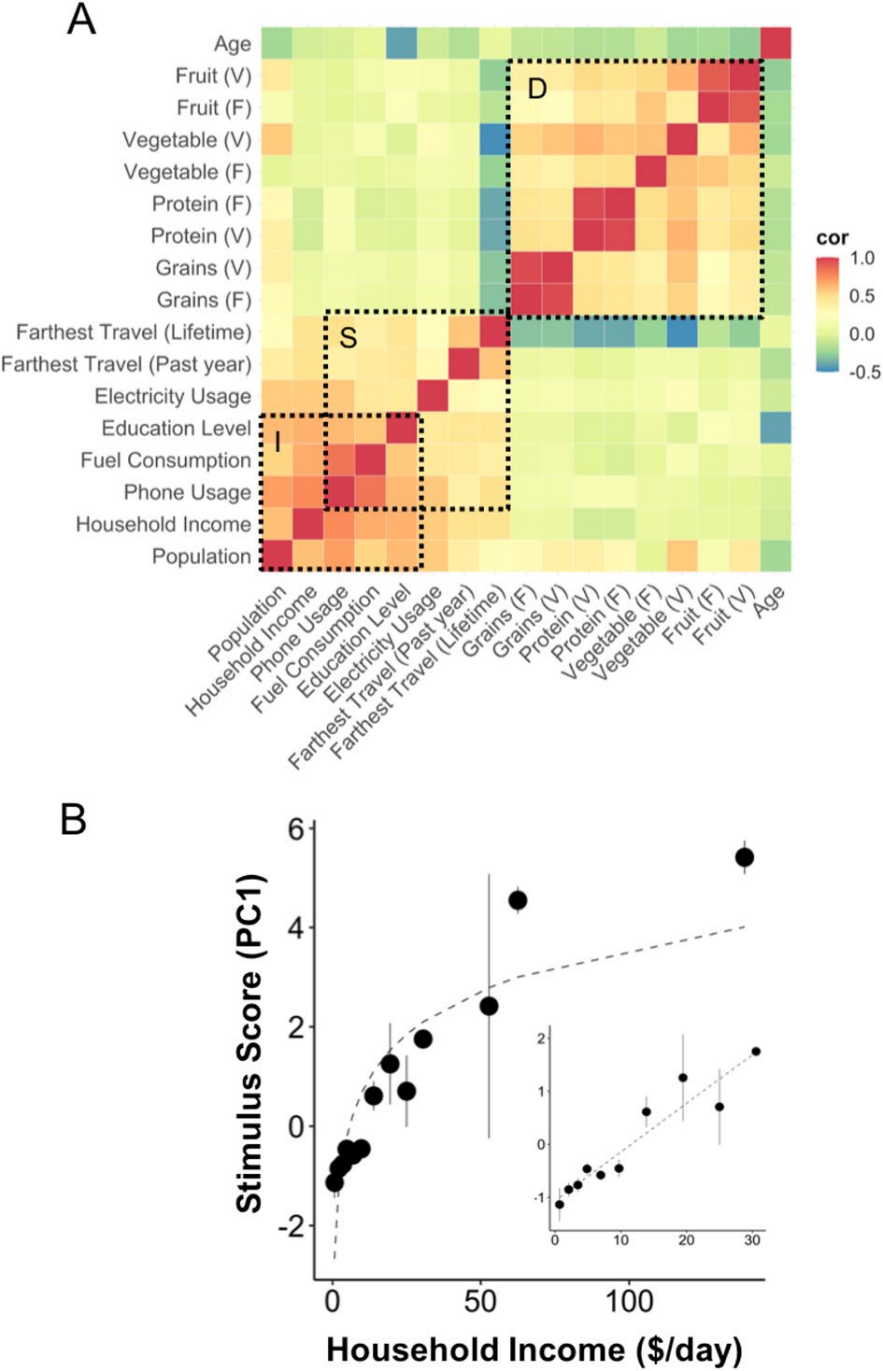
Survey factors and their inter-relationships. (**A**) Correlation matrix of all survey factors. Boxes marked D and S represent diet and stimulus factors respectively. Box marked I represents income and stimulus factors with the most significant correlation. (**B**) Composite PC1 score for stimulus consumption scales logarithmically with household income (R^2^ 0.7, *P*_ANOVA_ 2.6E-58). Inset: expansion of data in the range up to $30/day with linear fit (R^2^ of linear fit 0.86, *P*_ANOVA_ 6.9E-09).

**Figure 3:**
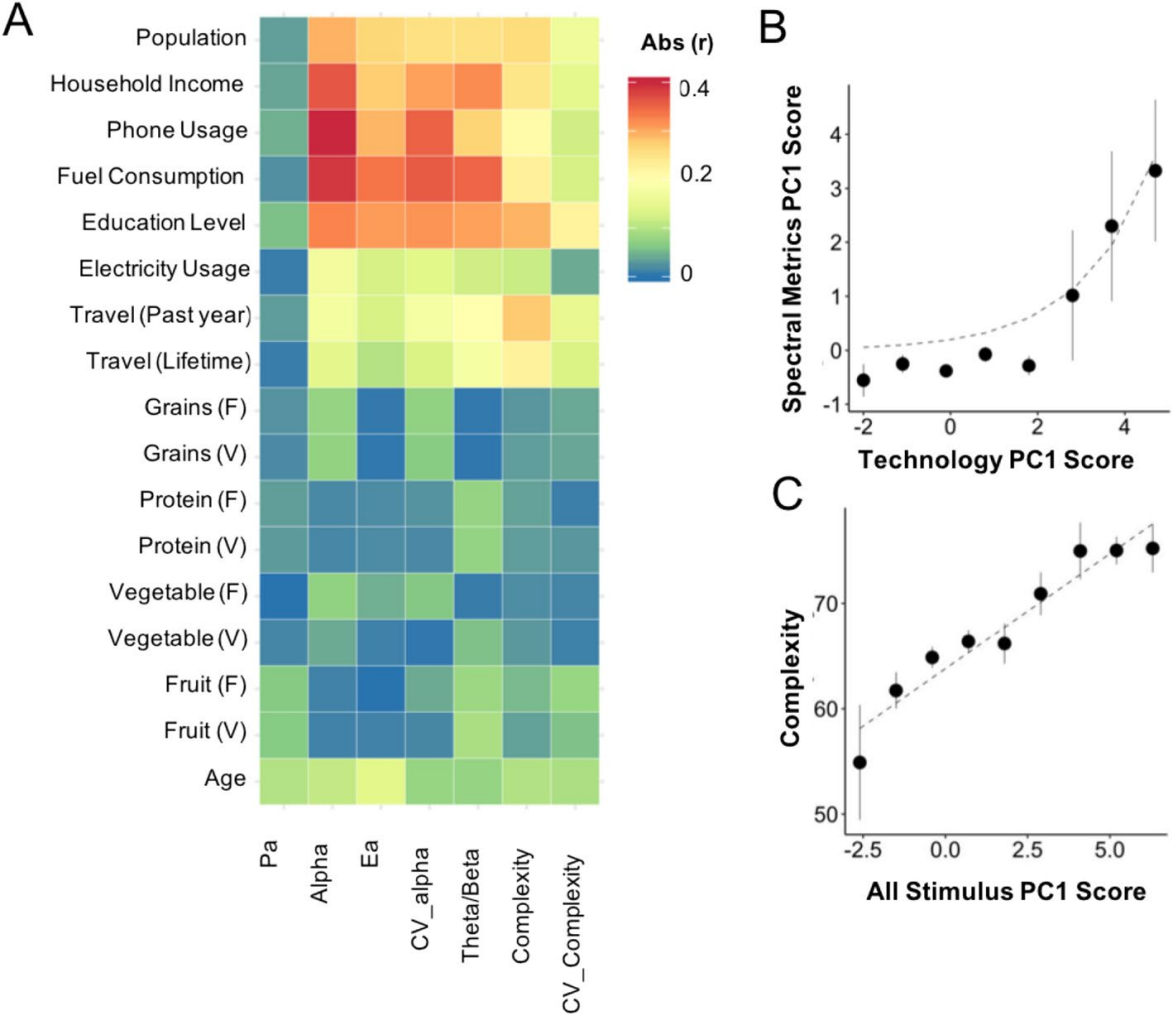
Correlations between EEG metrics and survey factors. (**A**) Correlation matrix of EEG metrics (columns) and input factors (rows) shows high correlation of stimulus factors but not diet factors to both spectral metrics and complexity. All r- and *p*-values shown in Supplementary Table 5. (**B**) Composite principal component (PC1) score for all spectral (energy) metrics plotted against composite technology PC1 score (phone, fuel, electricity) (R^2^ exponential fit 0.97, *P*_ANOVA_ 1.5E09). (**C**) Complexity plotted against composite PC1 score for stimulus consumption (R^2^ linear fit 0.91, *P*_ANOVA_ 8.9E04).

The Principal Component Analysis (PCA) was done in *R* using the FactomineR PCA. The component scores for the EEG, stimulus factors and diet factors of each individual were calculated by multiplying the contributions of each element by the unscaled individual element and then summing across the score of all components (Figs. 2B and 3B, C; Circle of correlations Supplementary Fig. 3, Eigenvalues Supplementary Table 6). For those factors with skewness > 3 (Supplementary Tables 1, 2), the values were log transformed using either a base 2 or base 5 to maximize the number of contiguous nonempty log bins. PCA was also done without Travel which is an arbitrary ordinal grouping and therefore may distort the results. However no significant difference was found (Supplementary Fig. 6; Table 2). Trends were computed on the bin means (Table 2) and also on the un-binned data as described in 2.8 (Supplementary Table 8).

**Table 2:** PCA based comparisons.

Survey factor	EEG metric/factors	Best fit	R2 (means)	F.ANOVA	*P*_ANOVA_	*p* Value best fit
Household income	Stimulus consumption PC1	Logarithmic	0.86	41.5	2.60E–58	5.43E–04
Household income (upto $30/day)	Stimulus consumption PC1	Linear	0.9	6.78	6.90E–09	1.21E–04
Household income	Diet consumption PC1	Exponential	0.77	1.24	2.60E–01	8.20E–01
Stimulus consumption PC1	Spectral metrics PC1	Linear	0.74	7.42	4.30E–09	1.62E–05
Stimulus consumption PC1	Complexity	Linear	0.92	3.66	4.20E–04	3.00E–03
Technology consumption PC1	Spectral Metrics PC1	Exponential	0.97	7.76	1.50E–09	3.20E–04

### Comparison of low-stimulus and high-stimulus groups

**Figure 4:**
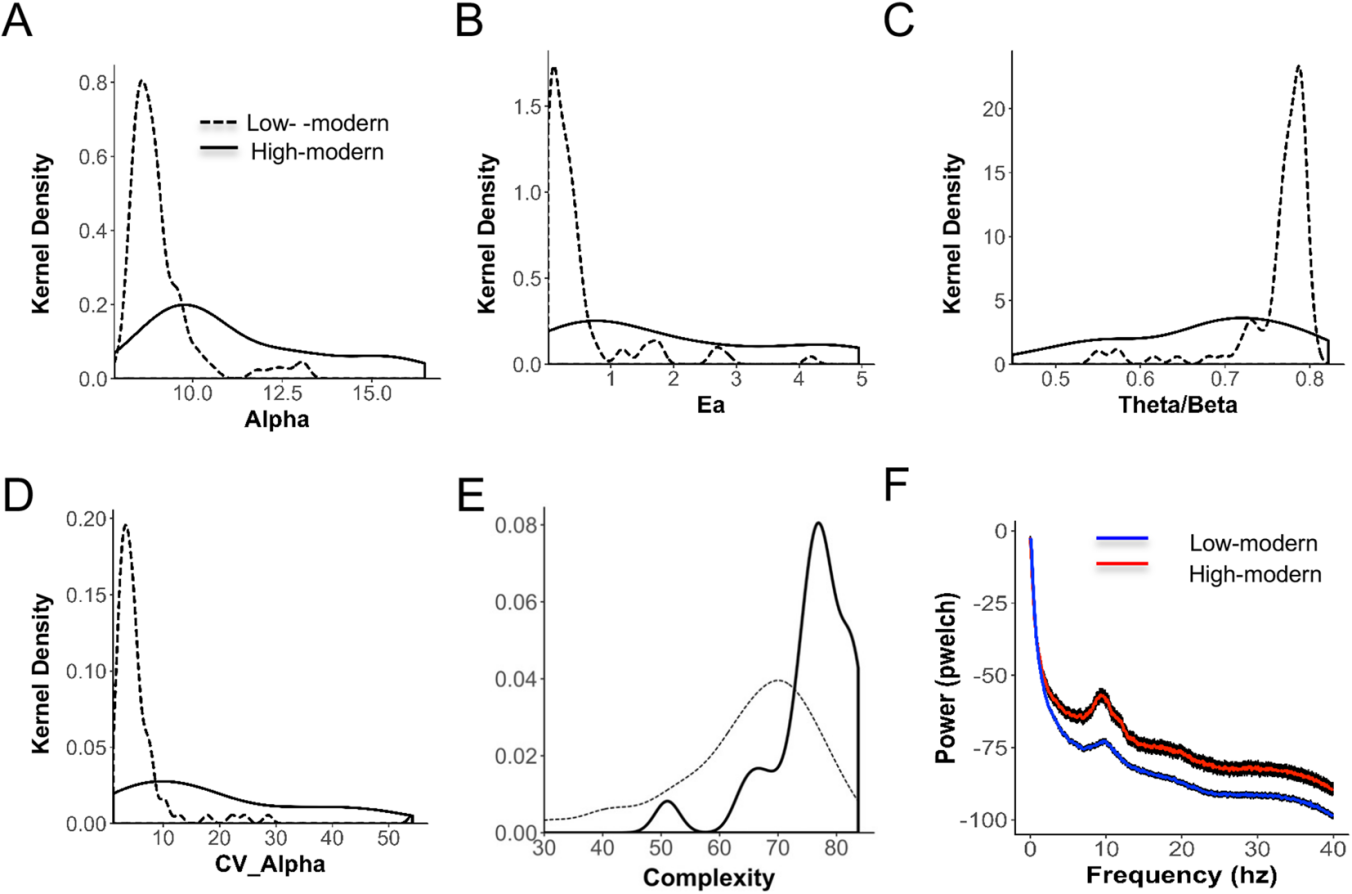
Differences between low-stimulus and high-stimulus groups. (**A**) Densities of Alpha (relative alpha power) for low-stimulus and high-stimulus groups shows 1.23 × difference in means with *P* < 2.9E-04 (K–S test). (**B**) Densities for Ea (peak alpha energy) shows 4.8 × difference in means with *P* < 3.9E-05 (K–S test). (**C**) Densities for CV_Alpha shows 3.5 × difference in means with *P* < 5.8E-04 (K–S test). (**D**) Densities for Theta/Beta ratio shows 1.13 × difference in means (here decrease, *P* < 5.4E-05 (K–S test). (**E**) Densities for Complexity shows 1.17 × difference in means with *P* < 7.5E-05 (K–S test). (F) Average power spectrums of low- and high- stimulus groups. Error bars: average spatial variability across individuals.

We compared EEG metrics between groups that represented the two extremes of stimulus consumption in the sample (Fig. 4 and Table 3). The ‘low-stimulus’ group had fuel spend < $15/mo, phone < $3/mo, a primary education or less and corresponded to income of < $10/day (N = 59, average age 44 ± 8 years, 55% Male). The ‘high-stimulus’ group (N = 29; average age 37 ± 10 years, 85% Male) had fuel spend > $30/mo phone spend > $25/mo and a college education or beyond, corresponding with income of > $50/day. Statistics used to compare these two groups included the one-sided *t*-test for samples of unequal variance and the non-parametric Kolmogorov–Smironov (K–S) test. A metric was considered statistically significant between the two groups when both of these *p*-values were < 0.01 (assuming a Bonferroni correction factor of 5 equal to the number of metrics compared). Note that there was no difference in any EEG metric between stimulus matched age or gender groups (Supplementary Table 11).

### Correlations and trend statistics

Pearson’s correlations and associated p-values were computed between each of the metrics (Fig. 1B; Supplementary Table 3) and survey factors (Fig. 2A; Supplementary Table S4) and between Factors and EEG Metrics (Fig. 4A; Supplementary Table S5). Significance levels were adjusted using a Bonferroni correction based on the number of comparisons.

**Figure 5:**
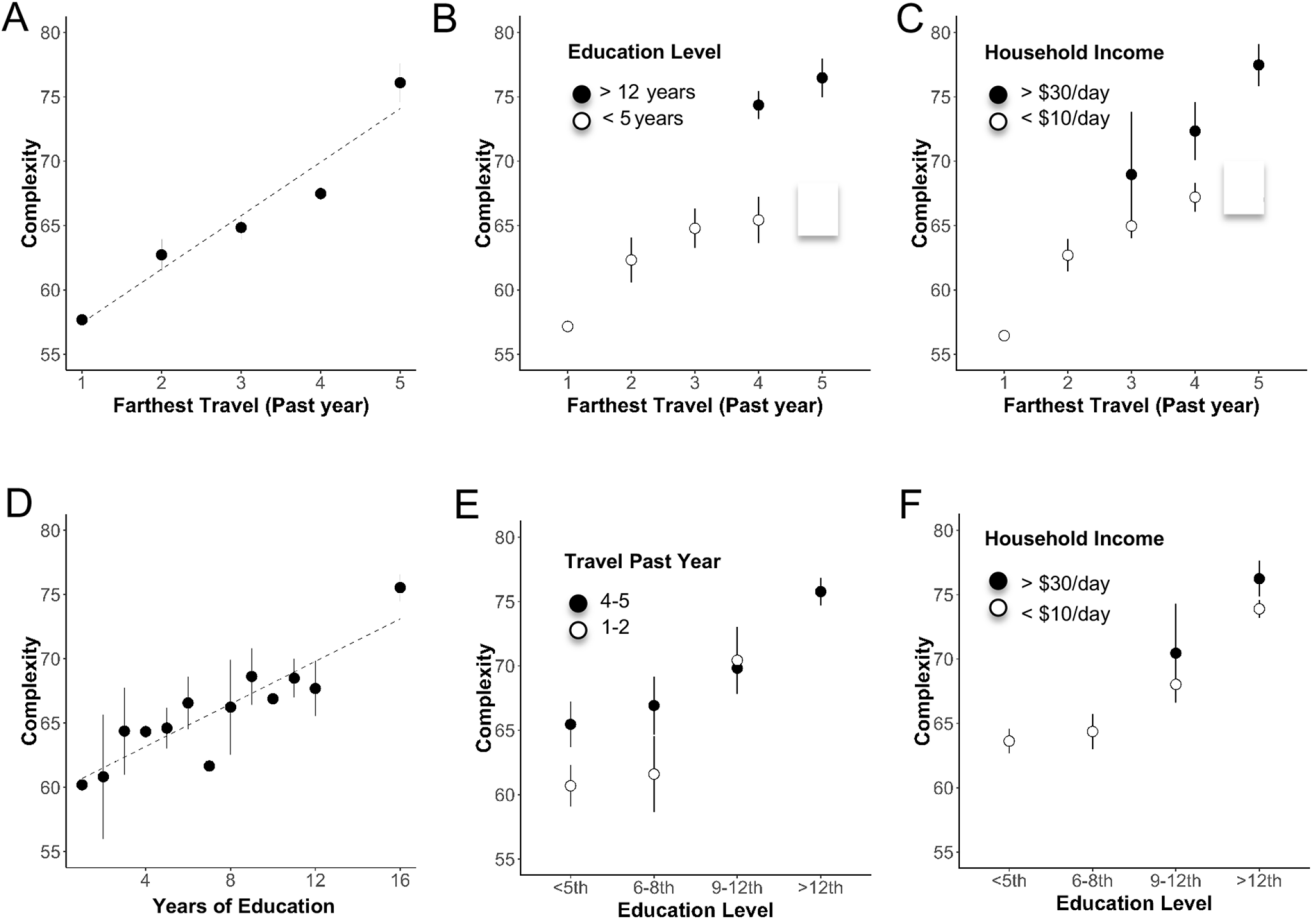
Relationship between education, travel and EEG complexity. (**A**) Mean Complexity ± SEM as a function of Travel (past year). Note all statistics relevant to figure 5 are shown in Table 4. (**B**) Complexity versus Travel for low and high education groups. (**C**) Complexity versus Travel for low- and high- income groups. (**D**) Complexity as a function of Education level. (**E**) Complexity versus Education for low and high travel groups. (**F**) Complexity versus Education for low and high income groups.

**Figure 6:**
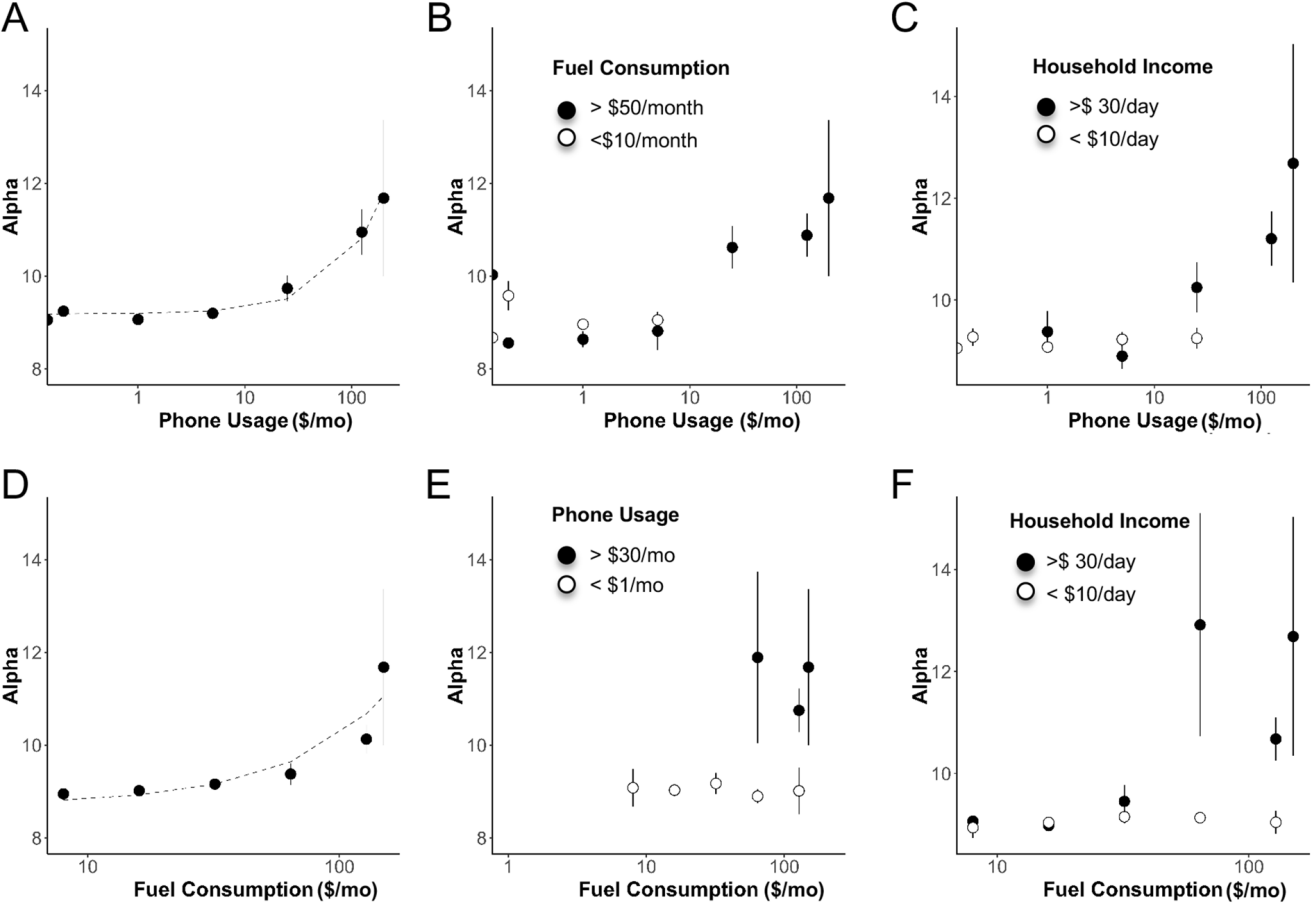
Relationship between phone, fuel and spectral metrics. (**A**) Mean Alpha ± SEM (where Alpha is relative alpha power) as a function of Phone usage (axis shown in log scale). (All statistics Table 4; N values Supplementary Table 9). (**B**) Alpha versus Phone Usage for low and high fuel consumption groups. (**C**) Alpha versus Phone Usage for low- and high- income groups. (**D**) Alpha as a function of Fuel Consumption (**E**) Alpha versus Fuel Consumption for low and high phone usage groups. (**F**) Alpha versus Fuel Consumption for low and high income groups.

**Figure 7:**
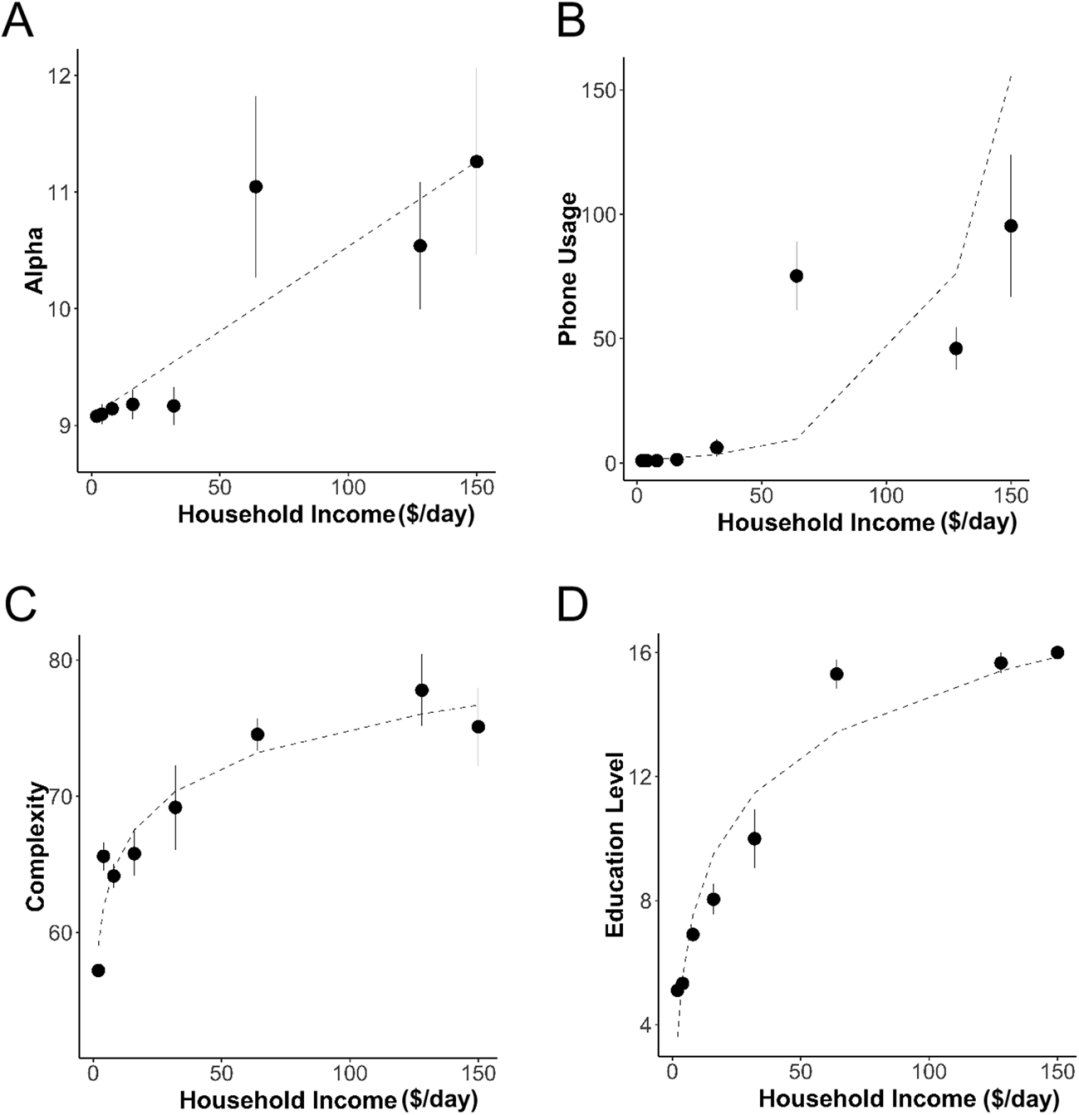
Relationship between income and key EEG features. (**A**) Alpha ± SEM as a function of Household Income. (**B**) Phone Usage as a function of income tracks pattern of Alpha as shown in A. (**C**) Complexity as a function of Household Income. (**D**) Education as a function of Household Income tracks pattern of Complexity as shown in C.

The trend of the relationship between each EEG metric and each survey factor was then determined using multiple statistics. First, we computed the ANOVA F-stats and *p*-values based on bins along the dimension of survey factors, where significance was determined after correcting for multiple comparisons (Supplementary Table S7). We then used multiple statistics to determine if there was a trend in the data (Supplementary Table S8). For each pair of EEG metric and survey factor we used the Mann–Kendall test to determine the significance of a linear trend. We also utilized the ordinary least squares (OLS) regression to compute the R^2^ and p-value of the each of linear, logarithmic and exponential trends. We similarly estimated the F-stat, R^2^ and *p*-values using the Theil–Sen robust regression estimator which is free of the assumption of a normal distribution and is not sensitive to outliers. The most significant trends by multiple estimations are shown in Table 4 and selectively in Figs. 5, 6 and 7. For each such trend shown in the Figures, the bin sizes were determined such that the first and last bins, where the number of participants was typically fewer, had at least 5 data points, (N values Supplementary Table 9).

### Density plots

Density plots in Fig. 4 were created using a kernel density estimation with a gaussian kernel and smoothing parameter equivalent to the standard deviation of the kernel to provide a smooth view of the distributions of the two groups. All statistics were computed on the actual data.

## Results

Here we have examined how the complexity and energy of the EEG signal relates to household income and consumption of stimulus factors such as education, travel, phone and fuel spend as well as consumption of major food groups (Table 1).

### Input factors and their relationships

Figure 2 shows a clustered correlation matrix (r) of all of the diet, stimulus and demographic factors (r- and p-values shown in Supplementary Table 4). Stimulus factors (box marked S) were all correlated to one another as were diet factors (D) (r > 0.2, *p* < E−05 both cases; Bonferroni corrected significance threshold = 3.68E−04). In contrast 82% of comparisons between stimulus and diet factors were not significant (max r of 0.28). Household income was significantly correlated with all stimulus factors (r > 0.47, *p* < E−19) but was not well correlated with diet factors.

Within S, phone use was most tightly correlated with fuel consumption (*r* = 0.83, *p* = 1.4E−36) and education (r = 0.62, *p* = 7.8E−52), and more correlated to income than other stimulus factors (*r* = 0.76, *p* = 3E−52). Travel was the least correlated to income and other stimulus factors. This may be due to the ordinal grouping of travel but may also indicate that it is a choice behavior. Finally, age, which was similarly distributed within each income group, was largely uncorrelated with most stimulus and diet factors, although strongly negatively correlated with education (*r* =  − 41, *p* = 5.1E−16). This likely reflects the increasing access to secondary and post-secondary education over the past few decades in India.

We next compared how overall consumption of stimulus and diet factors changed with income (Fig. 2B, Table 2). To do this we constructed composite stimulus and diet consumption scores for each individual based on the first principal component (PC1). The sample was then binned along the dimension of income and the average composite stimulus consumption score was computed for the individuals in each bin. The R^2^ was computed for the trend of the means. Statistical significance of the ANOVA and trends in the un-binned data was computed as described in Methods 2.8 (Table 2). The mean stimulus consumption score increased logarithmically with household income (R^2^ = 0.86, *P*_ANOVA_ = 2.6E-58; *P* < 5E−05 for all stats for log fit), where the steepest increase was in the range up to $30 per day (inset: linear fit R^2^ = 0.90; *P*_ANOVA_ = 6.9E-09, *P* < 8.2E-06 all stats for linear fit). In contrast there was no clear relationship between the composite diet score and income across this range (not shown, R^2^ = 0.77 exponential fit, *P*_ANOVA_ = 0.27).

### Correlations between survey elements and EEG features

We next looked at the correlations between each input factor and each EEG metric (Fig. 3, Supplementary Table 5). Income and stimulus factors, but not diet factors, showed high correlations with all EEG metrics except peak alpha frequency (Pa). Phone use had the highest correlations to energy metrics (Alpha, Ea and their corresponding spatial variability; r > 0.3, *p* < 1.6E−07; Bonferroni corrected significance level = 4.2E−05). Education was correlated with all EEG metrics (except Pa; r = −0.06, p = 0.23) and travel was most highly correlated with complexity (r > 0.3, *p* < 2E−09). Electricity had the weakest correlations. Stimulus matched gender groups were not different with respect to any EEG metrics (Supplementary Table 11). Age, was also not strongly correlated with any of the EEG metrics but weakly anti-correlated with Ea (r =  − 0.15, *p* = 3.7E−03), suggesting a decline in alpha oscillation strength with age, consistent with reports in the literature^28,29^.

We next looked at trends of composite scores of EEG metrics as a function of composite scores of subsets of stimulus factors constructed using principal component analysis (see Fig. 3B, C, Table 2; also see Supplementary Fig. 5, Supplementary Table 6). The composite score of energy metrics (spectral metrics PC1 score) plotted as a function of the composite score of technology consumption (phone, fuel and electricity; (technology PC1 score)) shows an exponential increase beyond a certain technology consumption score (Fig. 3B; R^2^ = 0.97 exponential fit, *P*_ANOVA_ = 1.5E−09, *P* < 3.2E−04 all fit stats). Similarly, complexity plotted against the composite stimulus consumption score shows a largely linear pattern that appears to level out at the highest stimulus levels (Fig. 3C; R^2^ 0.92 linear fit, *P*_ANOVA_ 4.2E−05, *P* < 3.0E−03 all fit stats). Thus key aspects of the EEG signal increase systematically with stimulus consumption.

### Comparing high-stimulus and low-stimulus groups

Given the stronger relationships between stimulus factors and EEG metrics compared to diet factors, we focused our analysis going forward on these elements. To assess the magnitude of difference in the EEG metrics across the sample we compared the lowest and highest stimulus extremes of the population (Fig. 4) where low stimulus refers to minimal phone use and fuel consumption and no post-primary education (see Methods 2.7). In all cases people with the lowest stimulus consumption lived in small, remote villages with income < $10/day. High-stimulus refers to high phone and fuel consumption and post-secondary education, generally urban dwellers with incomes > $50/day. All EEG metrics (except Pa) showed highly statistically significant differences between the low and high groups (Table 3; most cases *p* < 0.001, K–S test).

**Table 3:** Comparison between low-stimulus and high-stimulus groups.

EEG metric	High-stimulus mean	Low-stimulus mean	High-stimulus SD	Low-stimulus SD	*P* value (ttest)	*P* value (KS test)
Alpha**	11.3	9.2	2.40	0.77	4.16E–04	2.96E–04
Ealpha (Ea)*	1.9	0.4	1.69	0.54	5.81E–04	3.98E–05
Theta/beta**	0.7	0.8	0.11	0.05	7.33E–04	5.40E–05
CV_Alpha**	19.2	5.5	15.52	5.37	3.97E–04	5.80E–04
Complexity**	75.6	64.6	7.44	12.12	1.38E–04	7.48E–05
CV_Complexity*	9.3	14.6	8.06	8.60	8.19E–03	2.35E–03
Peak alpha (Pa)	9.7	10.3	1.10	1.95	5.68E–02	5.07E–01

*Significant < 0.01; **Signifcant < 0.001.

Figure 4A–E show the densities (see Methods 2.9) of these EEG features for the high and low stimulus groups. Altogether the spectral differences translated to a shallower decay of the power spectrum (lower theta-beta ratio) and a larger alpha peak in the high-stimulus group (Fig. 4F) indicating a much higher energy signal. The black background to the lines in Fig. 4F represents the average standard deviation (SD) of the power spectrum across channels within each individual, demonstrating a higher spatial variability in the high-stimulus group. Correspondingly, the coefficient of variance of alpha across channels (CV_Alpha, Fig. 4D) was 3.8-fold higher in the high-stimulus group.

Overall the picture that emerges is an enormous magnitude of difference between the low- and high-stimulus groups in the energy and complexity of the EEG signal and their spatial variability, along with higher variability across individuals in the high-stimulus group.

### Relationships of EEG metrics to survey factors

We next examined the relationship of each individual EEG metric to each individual survey factor (demographic, stimulus and diet) (Table 4; key examples in Figs. 5A, D, 6A, D and 7) computing various statistics to determine significant trends (Methods 2.7; Table 4; all trends Supplementary Tables 7, 8). All EEG metrics (other than Pa) showed significant increasing trends (or decreasing in the case of theta/beta ratio) with increasing income and multiple stimulus factors. Phone usage, education and income had the most robust relationships to most EEG metrics, although alpha metrics were more related to phone usage. In contrast age did not have a significant relationship with any metric other than Ea and most diet factors did not have significant trends although the variety of fruit and vegetables had weak but significant trend relationships to alpha and the theta-beta ratio (although the correlations were not significant). The most significant trends (significant by all statistical tests and in correlations) are shown in Table 4. Subsequent sections focus on these specific trends between stimulus factors and EEG metrics.

**Table 4:** Significant trends between stimulus factors and EEG metrics.

	Survey factor	EEG metric	F.Anova	P.F.Anova	Mann–Kendall pval	Model	F.ols	p-F.ols	R2.ols	F.tsrob	p-F.tsrob	R2.tsrob
1	Household income*	Theta/beta	7.82	7.9E–09	0.01	Linear	38.68	3.1E–05	0.75	38.76	3.1E–05	0.749
2	Household income*	Complexity	5.4	6.8E–09	0.00	Logarithmic	31.19	8.8E–05	0.71	34.39	5.6E–05	0.726
3	Household income	Alpha	11.39	4.4E–13	0.05	Exponential	27.76	1.5E–04	0.68	5.13	4.1E–02	0.283
4	Household income*	Ealpha	5.84	2.0E–06	0.01	Linear	27.58	1.6E–04	0.68	23.88	3.0E–04	0.647
5	Education level*	Alpha	6.06	1.2E–09	0.01	Exponential	15.67	2.2E–03	0.59	5.99	3.2E–02	0.352
6	Education level	Ealpha	4.18	4.2E–06	0.01	Linear	25.83	3.5E–04	0.70	19.38	1.1E–03	0.638
7	Education level	Complexity	4.15	4.5E–06	0.00	Linear	43.15	4.0E–05	0.80	33.65	1.2E–04	0.754
8	Education level	CV_Alpha	5.64	7.3E–09	0.04	Exponential	8.38	1.5E–02	0.43	5.74	3.5E–02	0.343
9	Education level*	Theta/beta	4.63	5.6E–07	0.01	Linear	20.15	9.2E–04	0.65	8.09	1.6E–02	0.424
10	Phone usage*	Alpha	11.71	1.1E–11	0.00	Exponential	30.32	1.6E–06	0.40	25.67	7.0E–06	0.358
11	Phone usage*	Ealpha	6.7	1.3E–06	0.00	Linear	11.59	1.4E–03	0.20	21.24	3.3E–05	0.321
12	Phone usage*	CV_Alpha	9.4	2.2E–09	0.00	Linear	17.32	1.4E–04	0.27	27.10	4.4E–06	0.371
13	Phone usage*	Theta/beta	7.47	2.0E–07	0.00	Exponential	9.06	4.2E–03	0.16	21.69	2.8E–05	0.320
14	Phone usage*	Complexity	4.14	5.4E–06	0.00	Linear	8.07	6.7E–03	0.15	20.35	4.6E–05	0.311
15	Fuel consumption	Alpha	6.28	2.8E–03	0.00	Exponential	11.55	2.9E–03	0.37	5.88	2.5E–02	0.227
16	Farthest travel (past year)*#	Complexity	10.45	5.1E–08	0.03	Linear	42.54	7.3E–03	0.93	39.12	8.2E–03	0.929

*Additional models are also significant, #variable is an ordinal grouping with each group assigned an arbitrary number. Thus only the direction of trend is significant and not the particular model.

### Education, travel and EEG complexity

Complexity increased linearly with education (Fig. 5A; R^2^ 0.8, *p* < 4.5E-06) and farthest travel in the past year (Fig. 5D; R^2^ 0.93, *p* < 5.1E-08). Since education and travel are significantly correlated and with one another (*r* = 0.57) as well as with income (*r* = 0.64 and 0.48 respectively), we next considered whether they had independent contributions to complexity (Fig. 5). To do this we looked at complexity as a function of travel for those with low education (< 5th grade) and those with high education (> 12th grade) (Fig. 5B). Complexity in both the low and high education groups systematically increased with travel. However, those in the higher education group were higher overall suggesting that education may impact complexity over and above travel. Note that in the high education group of our sample, no individual had a travel category less than 4 and no individual in our low education group had a travel category of 5. We similarly looked at complexity as a function of travel for those with income < $10/day and those with > $30/day (Fig. 5C). Complexity increased linearly as a function of travel for both income groups, although the high-income group had greater complexity overall, suggesting that travel independently impacts EEG signal complexity.

Conversely, we plotted complexity as a function of education for low travel (groups 1 and 2) and those high travel (groups 4 and 5) (Fig. 5E), grouping education into primary (0–5 years), middle (6–8 years), high school (9–12 years) and college or above. Complexity increased with education for both low and high travel groups. However, there was no difference between the low and high travel groups at the high school level, which could be on account of the very small number of people with high school education who had low travel (N = 6). We similarly looked at complexity as a function of education for the income groups < $10/day and > $30/day (Fig. 5F). Once again complexity increased linearly as a function of travel for both income groups, with the high-income group having slightly but not substantially greater complexity overall. This data suggests that travel has an effect on complexity of the EEG that is independent of education and income. On the other hand, while education likely accounts for a large proportion of the income dependent change in complexity, it was more difficult to parse in part due to the close tracking of educational attainment with income. Note that complexity increased logarithmically with income (Fig. 7C) which closely mirrored the relationship between education and income (Fig. 7E).

### Phone, fuel and spectral features

We next looked at the relationship between phone use, fuel consumption and the alpha component of the power spectrum (Fig. 6). Here we see an exponential increase in Alpha with phone usage beyond ~ $30/month and fuel consumption of ~ $50/month (Fig. 6 A, D). The pattern was virtually identical for Ea and CV_Alpha (not shown).

Given the high correlation between phone usage and fuel consumption (r = 0.83) we next looked to see whether it was possible to parse out their differential relationships. We first assessed Alpha as a function of phone usage for low and high fuel consumption groups (< $10/month and > $50/month) (Fig. 6B). The high fuel consumption group mirrored the overall trend. However the low fuel consumption group did not have phone usage beyond the threshold of change, offering an inadequate comparison. Conversely, we looked at Alpha as a function of fuel consumption for two groups of phone usage (< $1/month and > $30/month) (Fig. 6E). There was a clear lack of change in Alpha in either group for any level of fuel consumption. However, Alpha was uniformly larger in the high phone usage group for all levels of fuel consumption. This suggests that the relationship between EEG spectral/energy features and technology are largely dominated by phone usage, while the relationships with fuel consumption are indirect reflections of its correlation to phone use.

We next looked at changes in Alpha as a function of phone usage for two income groups (< $10/day, > $30/day) (Fig. 6C). Phone usage in the low-income group was largely < $7/month. Furthermore, there was no change in Alpha across this range. In contrast, Alpha increased as a function of phone use beyond $30/month for the high-income group, as did its variability (indicated by the growing error bars). In contrast, while there was a wide range of fuel consumption in the low income group (up to $120/month for those whose livelihoods were in transport and delivery), there was no change in Alpha at any level of fuel consumption (Fig. 6F). We note further that the pattern of change of Alpha with income (Fig. 7A) mirrored the pattern of phone use with income (Fig. 7B). Thus phone use may be a key driver of changes in spectral characteristics or energy in a threshold dependent manner.

## Discussion

### Stimulus dependent divergence of human brain physiology

Numerous social and technological innovations from institutionalized education, electricity, cell phones and motorized transport have profoundly changed the rate and scope of human stimulus exposure over the past 200 years. While the disparities in access have been discussed widely, their impact on brain physiology has not been previously considered. In this study we have identified a significant relationship between income, consumption of the major stimulus expanding factors of education, travel, and phone use, and the energy and complexity of the resting state EEG signal. The most shocking aspect of these results is the immense magnitude of the differences in brain physiology between the two extremes of humanity—those still living in relatively premodern low-stimulus conditions with no more than a primary education and no phones, compared to college educated, digitally connected city dwellers. Given the context of the literature one would expect some differences to arise in overall brain activity as the stimulus environment changes. However, differences on the order of threefold or more, as we have shown along some dimensions, are unexpected. As a point of reference, differences in physiological characteristics associated with mental illness tend to be on the order of 20–35%^30^.

These findings demonstrate that we have not fully appreciated the immense capability of technologies and other changes to the stimulus environment to alter our brain physiology. As access to stimulus diverges across humanity, our findings also call into question the concept of a prototypical human brain, an idea that has dominated the field of neuroscience, suggesting instead that brain physiology must be viewed in the context of its stimulus exposure.

### Lack of effect of diet factors

Both diet and sensory stimulii of any kind have the potential to influence the wiring and resulting physiology of the brain. Proteins and carbohydrates provide the raw material and energy for all key cellular processes and low caloric intake or access to protein rich food can significantly impact the brain’s capability for wiring and plasticity. In this respect caloric malnutrition has been associated with reduced capacity to learn in children^31–34^. Likewise, fruit and vegetables deliver key micronutrients that play a role in various aspects of neuronal physiology from axonal outgrowth to synaptic plasticity^35–38^ and micronutrient deficiencies have been shown to impact cognitive and mental health outcomes^36,39,40^. While the variety of fruit and vegetables had directional trends with spectral metrics, they were not as significant. This is likely due to the lack of significant differences in the consumption of broad food groups across the wide income range that we looked at. Such gross differences in access to food groups are likely to be most apparent closer to the poverty line of $2 per day and not in higher income ranges where discretionary spending is increasingly to acquire stimulus factors. However, it is also possible that diet affects different aspects of the brain than stimulus.

### Education, travel and signal complexity

Both education and travel alter the novelty of stimulus exposure but do so in fundamentally different ways. Education focuses on symbolic systems—letters and numbers and their subsequent application to knowledge acquisition, and contributes substantially to IQ and cognitive outcome^31^. In contrast travel is a complex interactive experience that requires planning and navigating new spatial environments, people, languages and cultures. Both were associated with significant gains in complexity of ~ 20 points (~ 30%) from the lowest category to the highest. Further, the evidence suggests that the increases in complexity with travel and education were largely independent, although parsing out the specific effects are challenged by the high correlations among all factors and calls for much larger scale studies. Nonetheless, our results lend to a hypothesis that the complexity of the signal may reflect the complexity of learned associations in general, whether through symbolic systems or exploration of new environments.

### Phone use and spectral energy

Electricity, motorized transport and phones are major technologies that alter the rate and scope of stimulus exposure. We captured broad levels of use of these technologies by looking at the amount individuals spent on these technologies each month. While this cannot distinguish *how* people are using them, it provides a view of the consequences of access to these technologies at a population level. Spending on all of these technologies had high correlations to one another, and to income, and similar relationships with the spectral features, essentially a shift towards higher frequencies (a lower theta-beta ratio and stronger alpha oscillation) with increased consumption. There was also greater spectral variability across channels placed over different brain regions, and across individuals as consumption increased, likely reflecting differences in how these technologies were used.

Among the technologies, the evidence suggests that phone usage is the key driver of changes in spectral properties (Figs. 6, 7). The threshold where spectral features begin to change (> $10–$20/month) is at a level where there is a shift from the use of basic phones used for calling with prepaid sim cards to postpaid smart phone use with data plans. We hypothesize that the lack of effects at the low end of phone use could be an indication that it is not simply the ability to call and communicate verbally with more people that has altered brain physiology, but rather the enhanced ability to access information that a smart phone provides. Whatever the reason, it is imperative that we understand it further and call for attention to this as an important area of study.

### Possible mechanisms

Rodents raised in enriched stimulus environments display increased neurogenesis^41,42^, dendritic branching^9,10^ and enhanced cortico-cortical interactions^11,12^. The enriched stimulus environment enabled by modern tools may have similar effects resulting in a larger and more structurally complex network that produces more complex and variable dynamics with higher energy (i.e. skewed towards higher frequencies) and is visible as an increase in cortical volume and surface area. Credence for this hypothesis comes from studies in the United States demonstrating that childhood poverty has a negative effect on cortical volume^14^, surface area^15^, and gamma power in the EEG^16^. Conversely years of education have been shown to correlate positively with these structural aspects of the cortex^17,18^. It is also of interest to note that the relationship of income to surface area was logarithmic^15^ as was the relationship we have shown to EEG metrics. Post-mortem anatomical studies of people in low- and high-stimulus environments could reveal the extent of underlying structural differences.

### Implications for cognitive function

What do these findings mean for the health and functioning of the brain? Evidence suggests there may be substantial implications for cognitive health.

The complexity metric we have used here has not been extensively studied in relation to cognitive function. However it has been shown to be significantly correlated to performance on a Raven’s pattern recognition task^27^, and years of education, which correlates with complexity of the signal, is also associated with an increase in performance on cognitive tests^43,44^, suggesting that complexity may serve as an indicator of cognitive capacity.

The alpha oscillation, which is enhanced in high-stimulus populations, is hypothesized to play a role in selective attention and mental imagery^24–26,45,46^ and decrease in the eyes closed resting state with sleepiness and reduced alertness^47^. It also declines with age^48^ and is decreased in fragile X mental retardation syndrome^49^ Alzheimer’s and amnesic mild cognitive impairment^50^. Similarly, a higher theta/beta ratio, which reflects a steeper decay of the power spectrum and therefore lower signal energy, has been associated with symptoms of ADHD in children^51^ as well as sleep deprivation^52^. It will be interesting in future research to understand the relationships between stimulus consumption, sleep and cognitive outcome.

### Implications for mental health

A higher incidence of mental health disorders has been associated with both the fast paced stimulus environments of cities^53,54^ as well as lower socioeconomic status^16,55–57^. In India prevalence of mental health disorders decreases systematically with education from 11.8% for illiterate populations to 6.03% for college graduates and from 12.28% in the lowest income quintile to 8.76% in the highest income quintile^58,59^. However, it is unlikely that these changes in brain physiology primarily reflect differences in mental health status.

No individual disorder had prevalence over 5% other than alcohol dependence (11.5%) in Tamil Nadu^58,59^, which is not sufficient to drive the results. Significance levels between low stimulus and high stimulus groups persisted even when selectively removing the highest or lowest 5% or 10% of the data in one group or another (Supplementary Table 10). More significantly, depression (4.62%) is not associated with changes in alpha power or the theta-beta ratio^30^ and therefore cannot account for the spectral changes seen here. Also, alcohol dependence was 19× higher in males relative to females^58,59^ but none of the EEG metrics were significantly different between rural males and females ruling out alcoholism as an explanation. Altogether, no mental health disorder other than OCD had a similar pattern of spectral change or impact to alpha power in adults^30^. With < 1% OCD morbidity in Tamil Nadu^58,59^, and very small magnitude relative to the results here, it is an unlikely explanation. On the other hand, we note that alpha power, in addition to its correlation to attention and sleepiness is also correlated with higher trait anxiety^60^. Thus it will be of considerable interest to parse out the nature of the relationship between stimulus environment, brain physiology and cognitive and trait outcomes.

### Societal implications of diverging brain physiology

Much work has been done over the past decades to reduce global poverty and its impact on global health. A major effort has been around moving people above the poverty line, presently defined by the World Bank as $2 per day, determined as the minimum income to establish caloric parity and eliminate hunger^61,62^. While caloric parity may be achieved at $2 per day, our data indicates that reasonable parity in stimulus consumption was achieved only in the range of $30 to $50 per day. Correspondingly this was also the range where relative parity was achieved in the complexity and energy of the brain signal. At $30/day ($10,950/year) one can own a car and smart phone and afford school fees in India. However in India only about 0.5% have incomes above $30 per day^63^. In 2011 PPP terms, globally only 7% of humanity live on > $50 a day (considered high income) and 16% on $20-$50/day (considered upper-middle income) respectively. This highlights the importance of social policies around universal education and enabling infrastructure and the imperative for understanding causal relationships between stimulus factors, brain physiology and cognitive outcome.

### Limitations of the study

Our study raises several interesting and important hypotheses but has substantial limitations. First, it cannot provide direct insights into cause and effect. This is both a specific challenge of this study where there are no controls per se, but also a general challenge of studying natural human systems where individual behavior and environments cannot be dictated and controlled. Second, individual factors were gross approximations of complex underlying phenomenology. For instance, while more years of education may roughly approximate more knowledge, curriculum and quality of teaching can differ substantially such that two people with the same years of education may have had quite different knowledge exposure. Our measures of diet similarly captured only broad food groups that do not reflect the nutrient quality of the choices within those groups. A third significant limitation is the difficulty of parsing out the impact of individual factors given that most stimulus factors tended to move together. However, despite these limitations we bring to light a general phenomenon of wide divergence of human brain physiology that is systematically related to the stimulus environment, generating a number of hypotheses that warrant further study.

## Supplementary Information

Supplementary Information 1.

Supplementary Information 2.

Supplementary Information 3.
