# Author Correction: The impact of socioeconomic and stimulus inequality on human brain physiology

**DOI:** 10.1038/s41598-021-92234-8

**Published:** 2021-06-14

**Authors:** Dhanya Parameshwaran, S. Sathishkumar, Tara C. Thiagarajan

**Affiliations:** Sapien Labs, 1201 Wilson Drive 27th Floor, Arlington, VA 22209 USA

Correction to: *Scientific Reports* 10.1038/s41598-021-85236-z, published online 02 April 2021

The original version of this Article contained an error in the legend of Figure 4, where the minus sign was missing in the *P* values.

“Differences between low-stimulus and high-stimulus groups. (**A**) Densities of Alpha (relative alpha power) for low-stimulus and high-stimulus groups shows 1.23 × difference in means with *P* < 2.9E04 (K–S test). (**B**) Densities for Ea (peak alpha energy) shows 4.8 × difference in means with *P* < 3.9E05 (K–S test). (**C**) Densities for CV_Alpha shows 3.5 × difference in means with *P* < 5.8E04 (K–S test). (**D**) Densities for Theta/Beta ratio shows 1.13 × difference in means (here decrease, *P* < 5.4E05 (K–S test). (**E**) Densities for Complexity shows 1.17 × difference in means with *P* < 7.5E05 (K–S test). (F) Average power spectrums of low- and high- stimulus groups. Error bars: average spatial variability across individuals.”

now reads:

“Differences between low-stimulus and high-stimulus groups. (**A**) Densities of Alpha (relative alpha power) for low-stimulus and high-stimulus groups shows 1.23 × difference in means with *P* < 2.9E-04 (K–S test). (**B**) Densities for Ea (peak alpha energy) shows 4.8 × difference in means with *P* < 3.9E-05 (K–S test). (**C**) Densities for CV_Alpha shows 3.5 × difference in means with *P* < 5.8E-04 (K–S test). (**D**) Densities for Theta/Beta ratio shows 1.13 × difference in means (here decrease, *P* < 5.4E-05 (K–S test). (**E**) Densities for Complexity shows 1.17 × difference in means with *P* < 7.5E-05 (K–S test). (F) Average power spectrums of low- and high- stimulus groups. Error bars: average spatial variability across individuals.”

The original Article has been corrected.

