# Supplementary material for: The impact of socioeconomic and stimulus inequality on human brain physiology
Source: Sci Rep. 2021 Apr 2;11:7439. doi: 10.1038/s41598-021-85236-z (PMC8018967; doi:10.1038/s41598-021-85236-z)
Supplement: Supplementary file 2 — Supplementary Information 2. [file 41598_2021_85236_MOESM2_ESM.pdf]

A

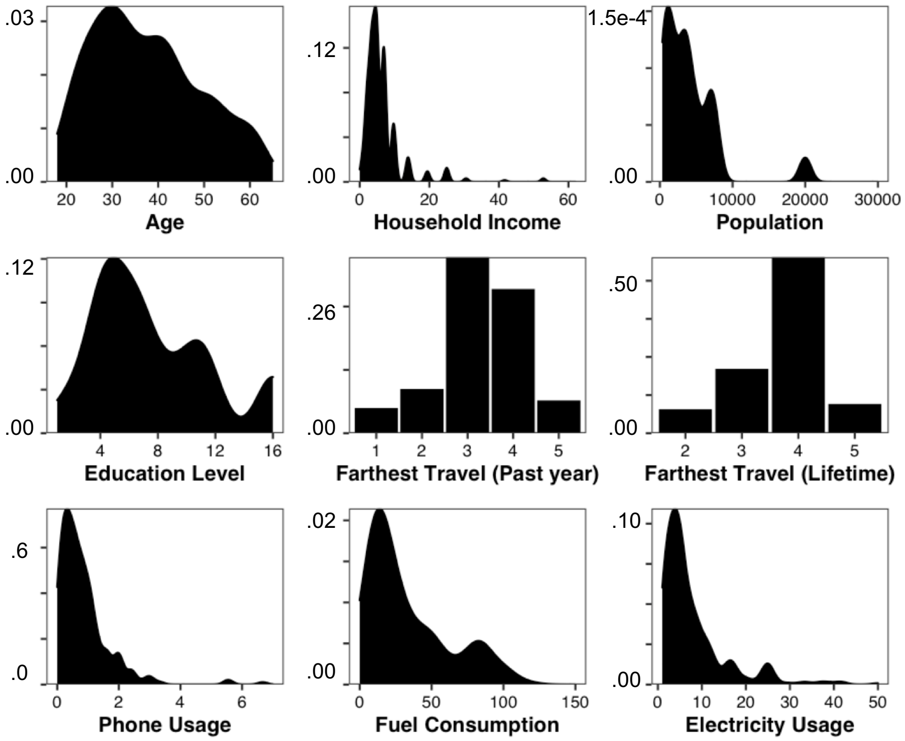

Histograms of stimulus factors. Household income, Phone usage, fuel consumption and electricity in $/month, Education level = years of education and Farthest travel represents ordinal categories.

Parameshwaran et al,
Supplementary Figure1

A

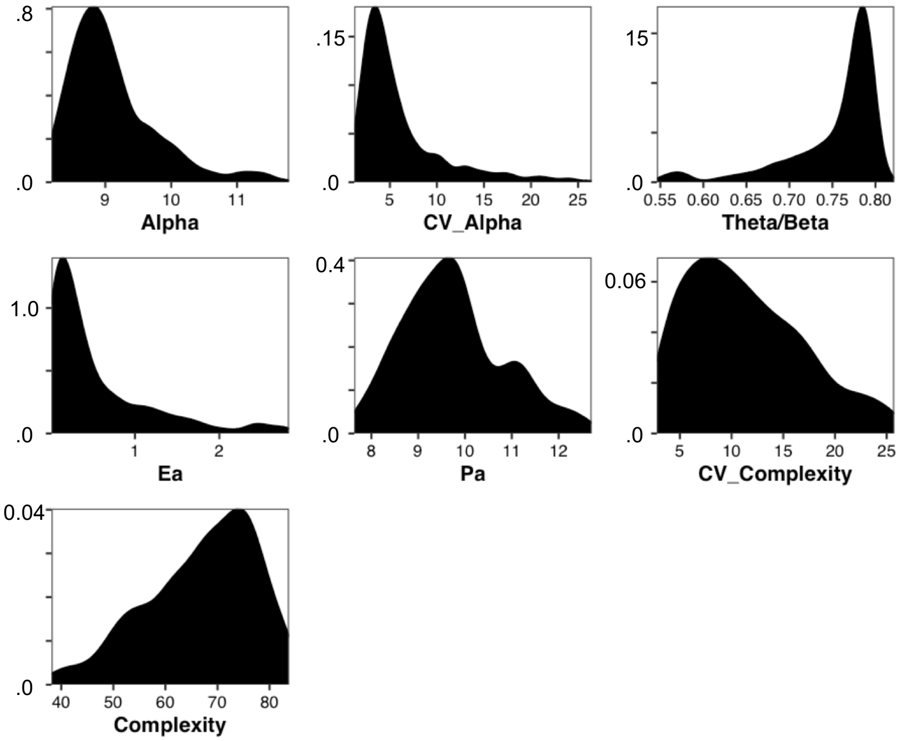

Histograms of EEG Metrics. All metrics are unitless or have arbitrary units (see Methods)

Parameshwaran et al,
Supplementary Figure 2

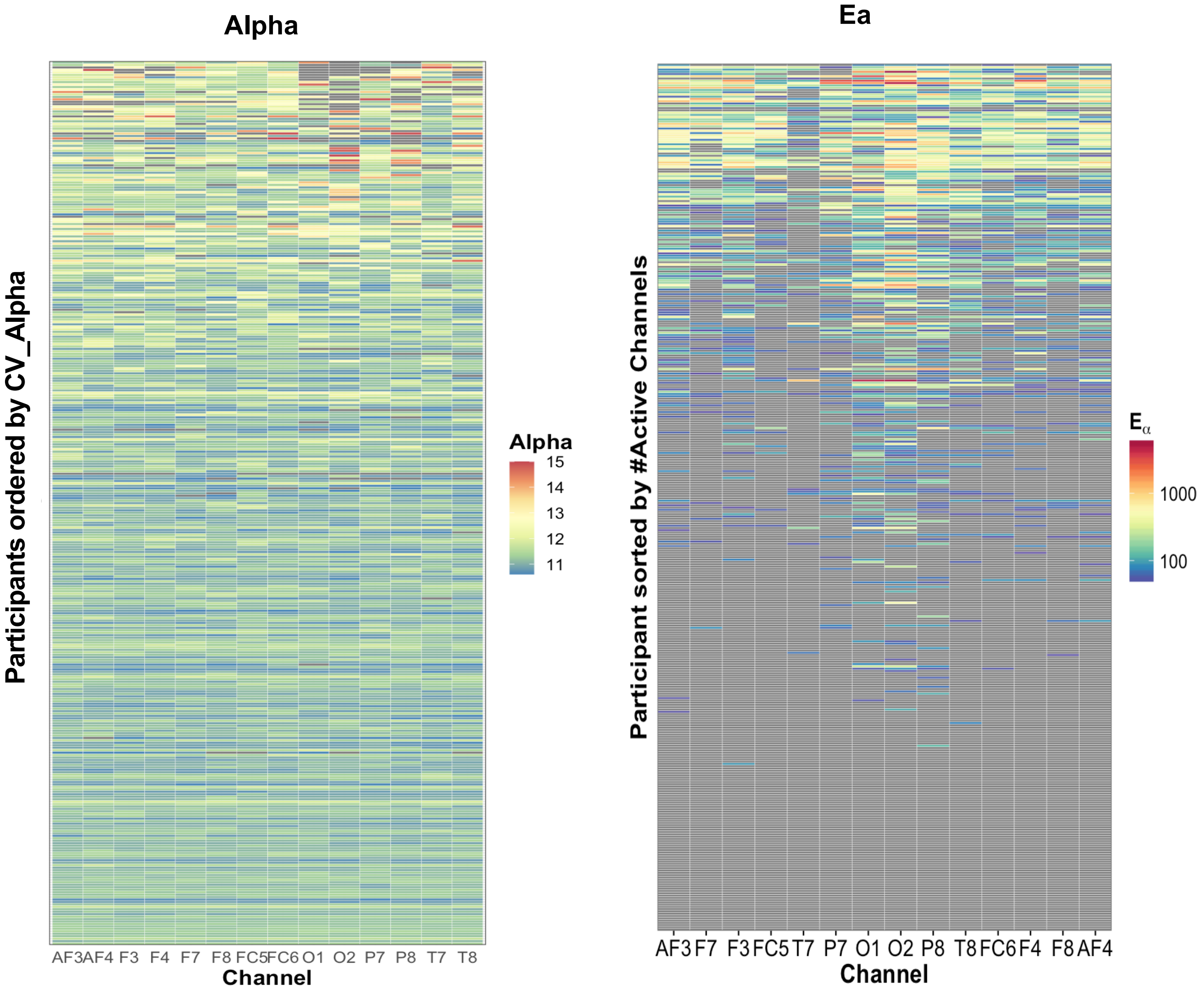

Alpha and Ea of subjects ordered by CV_Alpha (left) and no. of channels with oscillation peak (right).
Oscillation peak arises most frequently on channels O1 and O2.

Parameshwaran et al,
Supplementary Figure 3

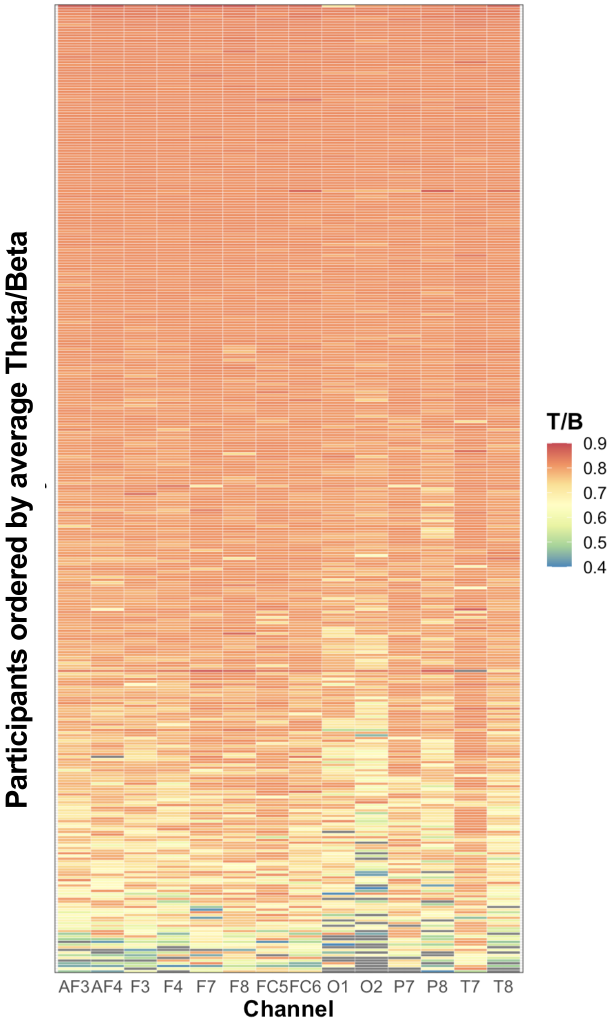

Theta/Beta ratio of subjects ordered by average Theta/Beta ratio. Channel wise differences suggest possible channel specific differences.

Parameshwaran et al,
Supplementary Figure 4

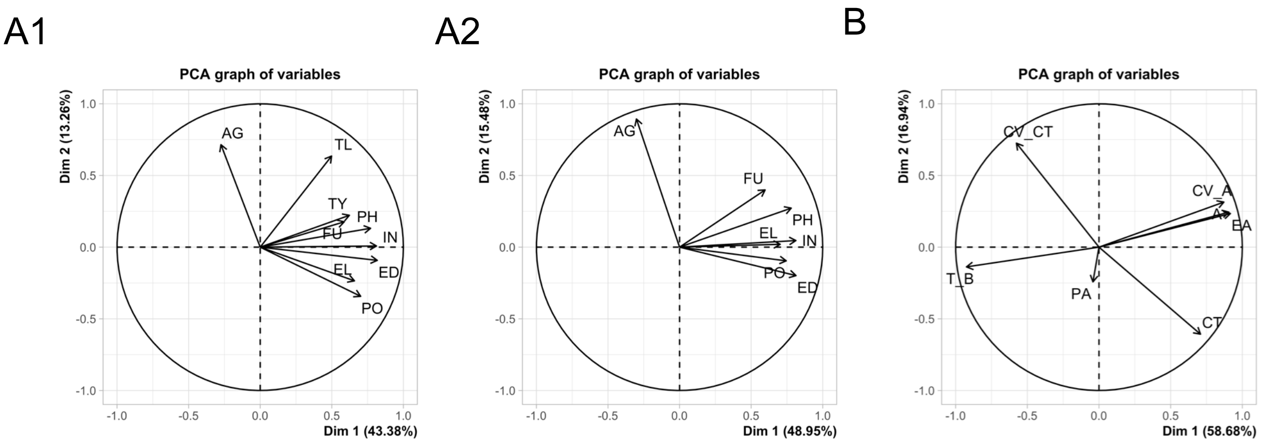

N=382

Circle of Correlations of first and second principal components for (A1) all stimulus factors
(A2) All stimulus factors excluding Travel variables and (B) All EEG metrics

Parameshwaran et al,
Supplementary Figure 5

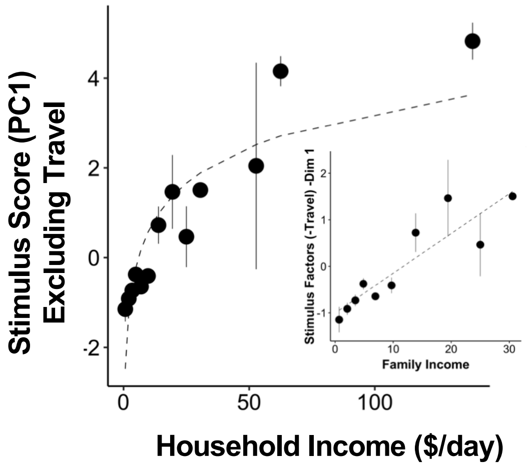

Stimulus score based on PC1 excluding travel as a function of household income ($/day).

Parameshwaran et al,
Supplementary Figure 6
