# Supplementary material for: The impact of socioeconomic and stimulus inequality on human brain physiology
Source: Sci Rep. 2021 Apr 2;11:7439. doi: 10.1038/s41598-021-85236-z (PMC8018967; doi:10.1038/s41598-021-85236-z)
Supplement: Supplementary file 3 — Supplementary Information 3. [file 41598_2021_85236_MOESM3_ESM.pdf]

Stimulus Consumption Questionnaire

*** If respondent refuses to answer any question please select/enter **RA** in the space. Do not leave it blank.***

Section 1: Contact Information

Respondent Name____

Address ____

Village/Town/City____

State _____________________________ Pin Code____________________________

Phone _______________________ Email _________________________________

Contact Preference Phone ____ Email _____

Section 2 Demographic Information

Year of Birth __________ Gender ___M ___F

Highest Level of Education [Drop Down – No School, 1,2,3,4,5,6,7,8,9,10,11,12,Diploma, College, Masters, PhD]

Monthly Household Income (include all family members contributing to the household): [Drop Down Bins of Rs. 2500 width]

Are you employed/working? ____ Y ____ N

If respondent answers yes.

What is your individual monthly income: [Drop Down Bins of Rs. 2500 width]

What is the type of Income (select all that apply) ___Daily Wages ____Salary ____Business Income

Section 3: Technology Ownership and Use

Select all technology assets that you own AND use

Transport

Do you have a motorized vehicle (bike, car, tempo): ____ Y ____N

If respondent answers yes:

How much do you spend on petrol each month: _________ Rs./month

Communication and Internet

Which of the following do you own and use?

__Basic Phone (including landline) ___Smart phone ___ Laptop ____Desktop ____Tablet/Phablet

___Wifi Router ___ Internet Connection.

If respondent has a phone:

Do you use prepaid or post paid phone service? ___Prepaid ___PostPaid

How much do you recharge/pay for your phone use per month? _______ Rs./month

If respondent has internet access:

How many **hours** do you surf/use the internet in **ONE WEEK**? ______**Hours/ week**

How long did you surf/use the internet yesterday? ____Hours

Energy usage

Do you have an active electricity connection? ___Y ___N

If respondent answers yes:

How many hours in a day do you have a power cut/outage (on average)? _______ hours/day

How much was your last electricity bill? _______ Rs. per month

Section 4: Travel

How long have you been living here ____ years ____months

If you moved here after the age of 17 what was the reason? ___Marriage ___Better Job ___Start Business ____Other

*Farthest location where……… **(Circle All Codes that Apply)***

Farthest Location where....	Native Village/ Town Only	Within State (<100 km)	Within State, (>100 km)	Another State (<100 km)	Another State (>100 km)	Abroad
Have Friends Living in	1	2	3	4	5	6
Have Family Living in	1	2	3	4	5	6
Have Travelled in the last one year to:	1	2	3	4	5	6
Have travelled in entire life to	1	2	3	4	5	6
Have lived for more than 1 month	1	2	3	4	5	6

What is the furthest place from here you traveled last year: ____

What is the furthest place from here you traveled in your lifetime: ____

Nutrition Section

Please select

- F for things you eat frequently (at least 4x per week)
- S for sometimes (1-3x per week, or daily but only when seasonal)
- R for Rarely (2x in a month or less)

	Food Type	Frequency	Did you eat this yesterday?	Do you get this in your village?
1	Rice/Idli/Dosa	F/ S/ R	Y/ N	Y/ N
2	Roti/Chappati	F/ S/ R	Y/ N	Y/ N
3	Sambar	F/ S/ R	Y/ N	Y/ N
4	Dhal	F/ S/ R	Y/ N	Y/ N
5	Rasam	F/ S/ R	Y/ N	Y/ N
6	Koozh	F/ S/ R	Y/ N	Y/ N
7	Kali	F/ S/ R	Y/ N	Y/ N
8	Vegetables	F/ S/ R	Y/ N	Y/ N

9	Fruit	F/ S/ R	Y/ N	Y/ N
10	Nuts	F/ S/ R	Y/ N	Y/ N
11	Milk	F/ S/ R	Y/ N	Y/ N
12	Curd/Buttermilk	F/ S/ R	Y/ N	Y/ N
13	Eggs	F/ S/ R	Y/ N	Y/ N
14	Fish	F/ S/ R	Y/ N	Y/ N
15	Chicken	F/ S/ R	Y/ N	Y/ N
16	Mutton	F/ S/ R	Y/ N	Y/ N
17	Shellfish (Crab, Prawns)	F/ S/ R	Y/ N	Y/ N

Which of these Fruits & Vegetables do you eat?

	**Fruits & Vegetables**	**Frequency**	**Did you eat this yesterday?**	**Do you get this in your village?**
1	Carrot	F/ S/ R	Y/ N	Y/ N
2	Tomato	F/ S/ R	Y/ N	Y/ N
3	Spinach (not murungakai leaves)	F/ S/ R	Y/ N	Y/ N
4	Bitter Gourd	F/ S/ R	Y/ N	Y/ N
5	Cabbage	F/ S/ R	Y/ N	Y/ N
6	Cauliflower	F/ S/ R	Y/ N	Y/ N
7	Potato	F/ S/ R	Y/ N	Y/ N
8	Brinjal	F/ S/ R	Y/ N	Y/ N
9	Ladysfinger	F/ S/ R	Y/ N	Y/ N
10	Beans	F/ S/ R	Y/ N	Y/ N
11	Capsicum	F/ S/ R	Y/ N	Y/ N
12	Onion	F/ S/ R	Y/ N	Y/ N
13	Murungakai	F/ S/ R	Y/ N	Y/ N
14	Yams (sepakezhangu)	F/ S/ R	Y/ N	Y/ N
15	Pumpkin/Gourds	F/ S/ R	Y/ N	Y/ N
16	Beetroot	F/ S/ R	Y/ N	Y/ N
